# Vehicle autonomous localization in local area of coal mine tunnel based on vision sensors and ultrasonic sensors

**DOI:** 10.1371/journal.pone.0171012

**Published:** 2017-01-31

**Authors:** Zirui Xu, Wei Yang, Kaiming You, Wei Li, Young-il Kim

**Affiliations:** 1 School of Electronic and Information Engineering, Beijing Jiaotong University, Beijing, China; 2 School of Mechanical, Electronic and Control Engineering, Beijing Jiaotong University, Beijing, China; 3 Electronics and Telecommunication Research Institute, Daejeon, Korea; IRCCS Istituto Auxologico Italiano, ITALY

## Abstract

This paper presents a vehicle autonomous localization method in local area of coal mine tunnel based on vision sensors and ultrasonic sensors. Barcode tags are deployed in pairs on both sides of the tunnel walls at certain intervals as artificial landmarks. The barcode coding is designed based on UPC-A code. The global coordinates of the upper left inner corner point of the feature frame of each barcode tag deployed in the tunnel are uniquely represented by the barcode. Two on-board vision sensors are used to recognize each pair of barcode tags on both sides of the tunnel walls. The distance between the upper left inner corner point of the feature frame of each barcode tag and the vehicle center point can be determined by using a visual distance projection model. The on-board ultrasonic sensors are used to measure the distance from the vehicle center point to the left side of the tunnel walls. Once the spatial geometric relationship between the barcode tags and the vehicle center point is established, the 3D coordinates of the vehicle center point in the tunnel’s global coordinate system can be calculated. Experiments on a straight corridor and an underground tunnel have shown that the proposed vehicle autonomous localization method is not only able to quickly recognize the barcode tags affixed to the tunnel walls, but also has relatively small average localization errors in the vehicle center point’s plane and vertical coordinates to meet autonomous unmanned vehicle positioning requirements in local area of coal mine tunnel.

## Introduction

Safe mining production is moving towards having few or no people working within the coal mines [1]. Autonomous driving of various vehicles in local areaof coal mine tunnels are important to achieve this goal [2–4]. To achieve autonomous driving, vehicles in local areaof coal mine tunnels require independent localization capability. Large-scale wireless sensor networks can be deployed in underground tunnels, which enable a vehicle to measure the distances to surrounding anchor nodes by using an on-board wireless sensor node, and then locates itself by using a triangulation algorithm [5,6]. The accuracy of this method is greatly affected by the geometry of the triangle. Due to a limited strip-shaped space of the underground tunnel, most locating triangles are flat, leading to relatively high localization errors. In addition, the energy consumption required by wireless sensor nodes prevents them from being used continuously for a long time [7–10]. Therefore, it is difficult to adopt this method in practice.

Vision sensors and relative image process technology are widely used in mobile object positioning due to their advantages such as accurate, low cost, easy usage and a relatively small environmental influence [11–16]. Currently, researches on autonomous positioning in a limited space based on vision sensors, mainly install sensors on the objects which require locating, e.g. the wheel robots. Some researchers concentrated on SLAM (simultaneous localization and mapping) algorithm and its improved algorithm. These algorithms realized the localization by collecting information of surrounding environment and building maps based on vision sensors [17–20]. The MCL(Monte Carlo localization) method, which locates robot position by using recursive estimation, is the most common localization algorithm used in SLAM. Thrun et al [21] proposed the Mixture-MCL, a robust algorithm based on the MCL, which integrates two complimentary ways of generating samples in the estimation. Some other researchers applied vision sensors to recognize landmarks real-timely and matched these landmarks with an on-board database to locate the object [22–24]. E Olson [25] proposed AprilTags Visual Fiducial System and it locates camera’s position by recognizing the AprilTags which were deployed in advance. However, the environments of local area for underground coal mine such as fully mechanized coal mining face may change frequently [26–28]. So, problems such as high complexity and low update rate of map exist for SLAM method. And the method of database landmark is limited by the need to build a huge database in advance.

We propose an autonomous localization method for coal mine vehicle that uses double vision sensors to recognize barcode tags on both sides of the tunnel walls with the help of ultrasonic sensors. Two cameras are fixed to the top center of the vehicle as the vision sensors, and the ultrasonic sensors are mounted below the cameras. Barcode tags containing their own position information in coal mine tunnel are affixed to the walls at both sides of the underground tunnel in pairs. When a vehicle travels in the tunnel, the on-board cameras collect the images of barcode tags on the walls at both sides of the tunnel, and get the barcode tags’ local area position information by recognizing the barcode on images. The distance from the upper left inner corner point of the feature frame of barcode tags on the walls at both sides of the tunnel to the center of cameras, i.e., the center of the vehicle, can be calculated from the ratio between the actual barcode tag width and its width in the CMOS (Complementary Metal Oxide Semiconductor) imaging plane [29]. Since ultrasonic sensors have some advantages such as small size, simple hardware, high accuracy of distance estimation and good performance in complex electromagnetic environment [30], the distance from the vehicle center point to the left tunnel wall is measured by the ultrasonic sensors which are mounted below the cameras. Finally, the 3D coordinates of the vehicle center point in the tunnel’s global coordinate system can be obtained based on the positional relationship between the projection of the upper left inner corner point on the camera CMOS imaging plane and the image center point. The localization method of vehicle autonomous localization in local area of coal mine tunnel based on vision sensors and ultrasonic sensors is a kind of geometric localization. Its calculation complex is much lower than the method of SLAM and it needs not to establish a landmark database in advance, which leads to a good positioning performance in real time.

The rest of this paper is organized as follows: a barcode tag that is suitable for autonomous localization in local area of a mine tunnel vehicle is designed, and a barcode locating and recognition algorithm is proposed in Section 2; a local area mine tunnel vehicle autonomous localization model and a localization algorithm are proposed based on a dual camera distance measurement and ultrasonic sensor distance measurement in Section 3; the proposed vehicle autonomous localization method in local area of coal mine tunnel is tested and evaluated in Section 4; and the relevant conclusion is presented in Section 5.

## Barcode tag design and recognition

**Fig 1:**
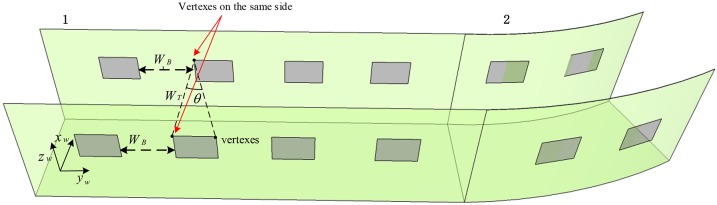
Illustration of the deployment of barcode tags in a coal mine tunnel.

Fig 1 shows an underground tunnel barcode tag deployment. It should be assumed that the tunnel walls are vertical on both sides, and the width of the tunnel which is represented by *W*_*T*_ is unchanged along underground tunnels in the local area of coal mine. The plane barcode tags are placed in pairs on the walls at both sides of the tunnel at interval distances of *W*_*B*_. *θ* is the angle between the lines from any barcode vertex to the two vertexes of the barcode tag of the corresponding pair on the wall on the opposite side of the tunnel, where the line between vertexes on the same side is perpendicular to the tunnel wall. A global coordinate system *o*_*w*_*x*_*w*_*y*_*w*_*z*_*w*_ is established in the tunnel as shown in Fig 1, where the transverse, longitudinal and vertical directions of the straight tunnel segment 1 are respectively the *x*_*w*_, *y*_*w*_ and *z*_*w*_ axes of the global coordinate system *o*_*w*_*x*_*w*_*y*_*w*_*z*_*w*_. Due to weak light in the underground tunnel, images collected by ordinary cameras are usually blurred. To effectively recognize the barcode tags affixed to the walls, infrared cameras are installed to ensure effective recognition of barcodes even when there is very weak visible light.

### Barcode tag design

UPC-A code (Universal Product Code-A) is a continuous barcode used to represent digital information and it can express a large amount of information. UPC-A code is mature, simple and easy to recognize. The barcode tags that are affixed to the tunnel walls use UPC-A code to represent their position coordinates in the global coordinate system. The barcode tag position coordinates are taken as the coordinates of upper left inner corner point of the feature frame of barcode tag.

**Fig 2:**
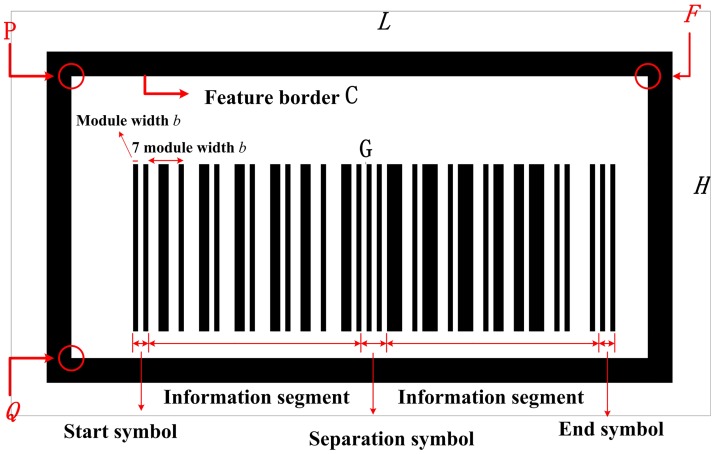
Design of barcode tag.

Fig 2 shows a barcode tag designed based on UPC-A code. The background of the barcode tag is a white rectangle of length *L* (m) and width *H* (m). The pattern on the white rectangle is divided into two parts. The first part is the feature frame C, a black frame with lengths of outer sides of *L*_*C*_ and *H*_*C*_ respectively and lengths of inner sides of *L*_*CI*_ and *H*_*CI*_ respectively. As shown in Fig 2, the feature frame C contrasts strongly with the white background, which makes it easy to be extracted by cameras from the white background. In addition, the white color both inside and outside of the frame C increases the accuracy of extraction under usually gloomy light conditions in the coal mine tunnels. In Fig 2, the red arrows mark the upper left inner corner point *P*, the lower left inner corner point *Q*, and the upper right inner corner *F* of the feature frame C.

The second part, G, is the main part of the barcode tag, i.e., the part from the leftmost black bar to the rightmost black bar surrounded by C, which consists of 59 black and white bars. The leftmost three bars (two black bars with a single white bar in between) are the start symbol, while the rightmost three bars are the end symbol, as shown in Fig 2. The five bars in the middle of G form the separation symbol, which is composed of three white bars with two black bars in between. The 24 bars between the start symbol and the separation symbol and the 24 bars between the separation symbol and the end symbol represent a 12-digit number, i.e., each digit is represented by four bars. In Fig 2, each digit represented by four bars has a width of 7*b* (*b* is module width), since each bar in the start symbol, the end symbol and the separation symbol has a width of *b*. Thus the width of the G is 95*b*. The first four digits, the middle four digits and the last three digits indicate the coordinates of the upper left inner corner point *P* of the feature frame C along the *x*_*w*_, *y*_*w*_ and *z*_*w*_ axes respectively in the global coordinate system *o*_*w*_*x*_*w*_*y*_*w*_*z*_*w*_. The last digit is a check digit which is the remainder of the sum of the first 11 digits divided by 10. This digit is used to check whether the coordinates of *P* recognized by the camera are correct. The global coordinates of *P* are encoded as a barcode using the coding rules of UPC-A barcode listed in Table 1. In the coding rules of UPC-A barcode, each digit of the 4-digit number in the encoding column indicates a single bar whose value is the multiple of the module width *b*. The different combination of 4 bars indicates different number, for example, “3211” indicates number “0” according to the coding rules of UPC-A barcode. During barcode recognition of an image captured by a camera, the same rules are used for decoding.

**Table 1:** UPC-A barcode encoding rules.

Number	Encoding	Number	Encoding
0	3211	5	1231
1	2221	6	1114
2	2122	7	1312
3	1411	8	1213
4	1132	9	3112

### Locating and recognition of barcode tags

**Fig 3:**
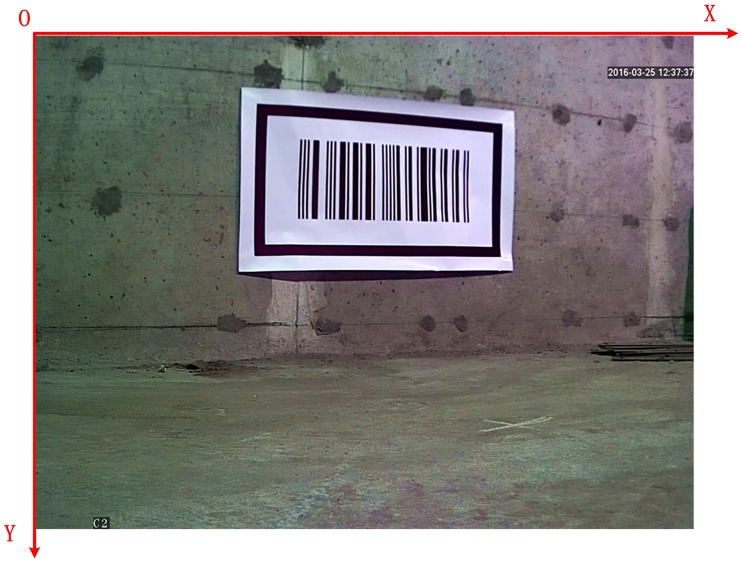
The original image *I*_*0*_ captured by a camera.

When a vehicle is traveling in the tunnel as shown in Fig 1, the on-board cameras capture the barcode tags affixed to the walls on both sides of the tunnel and images of some of the surrounding walls. If it is assumed that the resolution of the captured image on the CMOS imaging plane is *m* × *n*, i.e., the image pixels are arranged in *n* rows and *m* columns, then the pixel at row *i*(1 ≤ *i* ≤ *n*) and column *j*(1 ≤ *j* ≤ *m*) in the image can be represented by *p*(*i*, *j*). If it is assumed that the actual physical size of the image on the CMOS imaging plane is *M* × *N*, then each pixel has an actual side length *M* / *N*. Fig 3 shows the original image *I*_*0*_ including a barcode tag that is captured by a camera with a resolution of 1280×960. In this figure, a pixel coordinate system with the upper left corner point as the coordinate origin is established. In this pixel coordinate system, the positive direction of the X-axis is from the image’s left side to its right side, and the positive direction of the Y-axis is from the image’s upper side to its lower side, thus the pixel coordinates of *p*(*i*, *j*) will be X = *j*, Y = *i*.

After the original image *I*_*0*_ is obtained, the inner outline of the barcode tag feature frame C in the CMOS imaging plane can be extracted to locate the pattern of C within *I*_*0*_. Then the barcode tag recognition can be realized by scanning the barcode within the feature frame C.

#### A. Locating of the barcode feature frame C pattern

**Fig 4:**
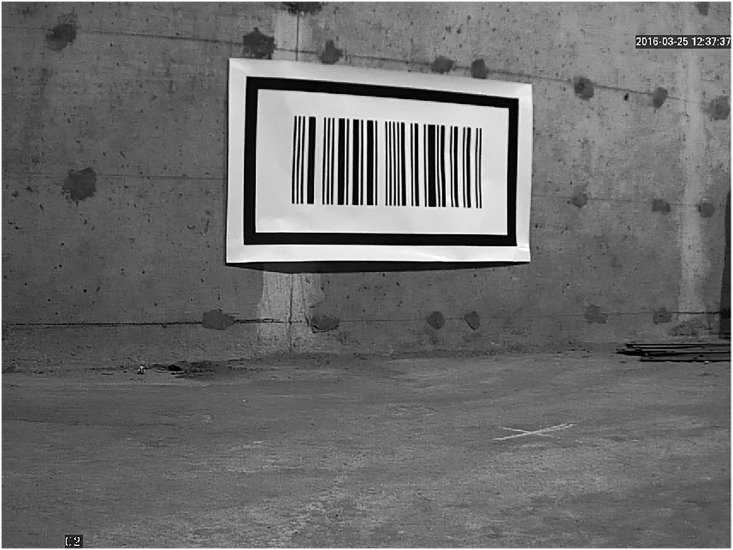
Gray scale image *I*_*1*_.

Fig 3 shows the original color image *I*_*0*_. Only black and white colors are useful to recognize C, so the color image *I*_*0*_ should be transformed to a gray scale image *I*_*1*_firstly, as shown in Fig 4. Each pixel in *I*_*1*_ can only have a pixel value corresponding to a brightness level from 0 to 255. A value of zero is used to indicate the lowest brightness (black color), while 255 represents the highest brightness (white color).

After obtaining the gray scale image *I*_*1*_, a binary image *I*_*2*_ containing only black and white pixels is generated using a binarization process. In image *I*_*2*_, the pixel values 0 and 1 represent the black pixels and the white pixels respectively. For the binarization process from a gray scale image *I*_*1*_ to a binary image *I*_*2*_, a threshold *t* is used to determine the pixel value in *I*_*2*_. A value 0 (black color) is given to pixel values less than *t*, and a value 1 (white color) is given to pixel values greater than or equal to *t*. To select the threshold value, OTSU algorithm [31–35] is used to maximize the variance of pixel values between different classes. Let *n*_*w*_(*t*) and *n*_*b*_(*t*) be the number of pixels that have values greater than or equal to the threshold *t*, or less than the threshold *t*, respectively, proportional to the total amount of pixels in *I*_*1*_. Let *μ*_*w*_(*t*) and *μ*_*b*_(*t*) be the average pixel values of the group of pixel values that are greater than or equal to the threshold *t* and the group of pixel values less than the threshold *t*, respectively. Let *μ*(*t*) be the overall average pixel value in *I*_*1*_, and *g*(*t*) be the variance between these two classes of pixel values. Then

$$\mu (t)=n_{w}(t)\mu_{w}(t)+n_{b}(t)\mu_{b}(t)$$ (1)

$$g(t)=n_{w}(t){\left(\mu_{w}(t)-\mu (t)\right)}^{2}+n_{b}(t){\left(\mu_{b}(t)-\mu (t)\right)}^{2}$$ (2)

**Fig 5:**
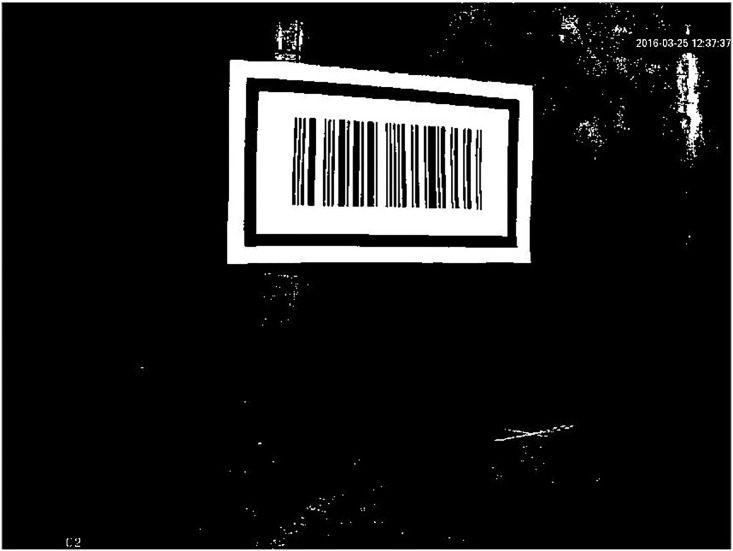
The binary image I2.

The threshold *t* is given a value from 0 to 255 in turn, and the value of *t* that gives the max (*g*(*t*)) is selected as the threshold *t*. The binary image *I*_*2*_ after the binarization process of *I*_*1*_ is shown in Fig 5, where threshold *t* is given a value of 153 based on the OTSU algorithm.

**Fig 6:**
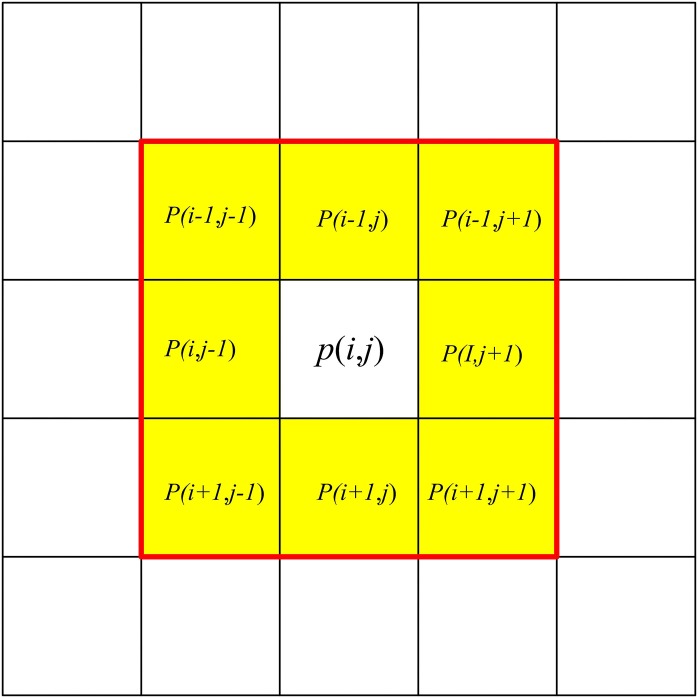
8 neighbor *N*_8_(*p*) of pixel *p*(*i*, *j*).

**Fig 7:**
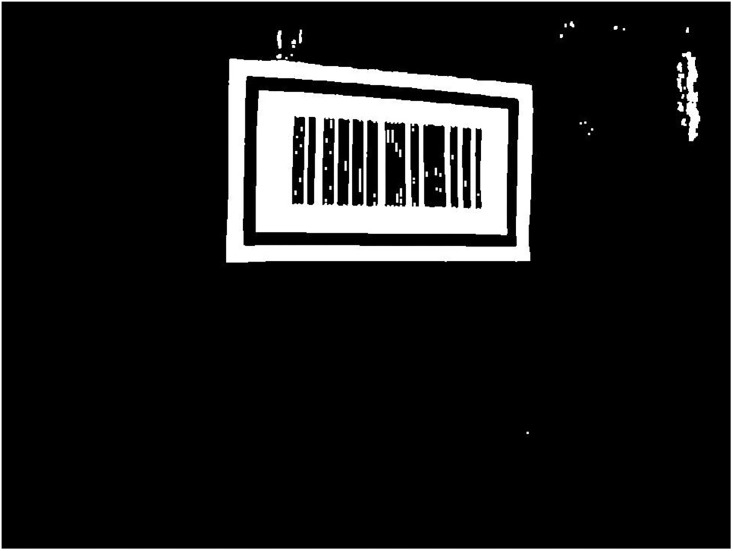
Resulting image *I*_*3*_ after opening operation.

As can be seen from Fig 5, after binarization, noise patterns such as the wall texture in the gray scale image *I*_*1*_ appear as a vast amount of tiny white porphyritic noise in the binary image *I*_*2*_. These noise patterns may misdirect the camera when locating the pattern of the barcode tag feature frame C and increase the required computation to extract inner outline of C. For this reason, an opening operation is used to eliminate small noise patterns in image *I*_*2*_ based on erosion followed by dilation. The erosion operation shrinks the boundaries of all white patterns in the image *I*_*2*_, thus eliminating small white noise patterns such as the wall texture. The dilation operation can then recover the remaining eroded boundaries of the white patterns in *I*_*2*_. When erosion and dilation are performed in the experiment, a 3 × 3 structure *A* is first created which can be considered as a binary image with 9 pixels of value 1. *A* is moved across image *I*_*2*_ and compared to each pixel *p*(*i*,*j*) and its 8 neighboring pixels *N*_8_(*p*), i.e. the 8 pixels around *p*(*i*,*j*) which are shown by the yellow area in Fig 6. During the erosion process, if *p*(*i*,*j*) and *N*_8_(*p*) all have the same value of 1 as *A*, then pixel *p*(*i*,*j*) is set to value 1; otherwise it is set to 0. During the dilation process, if any element of *p*(*i*,*j*) and *N*_8_(*p*) has a value of 1, then *p*(*i*,*j*) is set to value 1; otherwise it is set to 0. Fig 7 shows the image *I*_*3*_ after the opening operation of erosion and dilation. As can be seen from this figure, the opening operation eliminates most of the small white noise patterns such as the wall texture in image *I*_*2*_.

**Fig 8:**
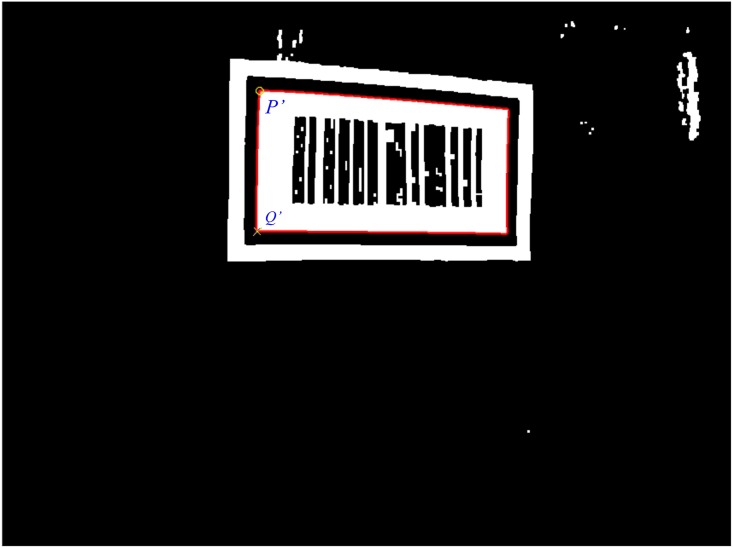
Located pattern of the barcode feature frame C.

After obtaining image *I*_*3*_ with most of the white noise patterns eliminated, the outlines of all the remaining white patterns can be extracted. During this extraction, for each white pixel *p*_*w*_(*i*, *j*), a check is performed on whether its 8 neighboring pixels *N*_8_(*p*) are all white. If any of 8 neighboring pixels is not white, then *p*_*w*_(*i*,*j*) is a boundary pixel. If all 8 neighboring pixels are white, then *p*_*w*_(*i*,*j*) is not a boundary pixel. The extraction is completed once the pixel coordinates of the boundary pixels have been determined. After all of the white pattern outlines in image *I*_*3*_ have been extracted, their areas are calculated. The outline of white pattern with the largest area is the barcode tag’s white background outline, and the outline of second largest white area is the inner outline of the feature frame C pattern which can be used to locate the black pattern of the feature frame C, as shown in Fig 8. The red line marks the inner outline of the feature frame C.

**Fig 9:**
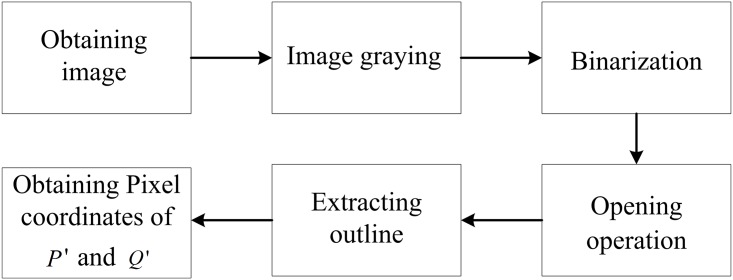
Locating process of the pattern of the barcode tag feature frame C.

Within the CMOS imaging plane, the projection points of the upper left inner corner point *P* and the lower left inner corner point *Q* of C are denoted by *P*′ and *Q*′, respectively. The length of *P*′*Q*′ is used to calculate the distance from *P* to the camera center point, i.e., the center point of the vehicle in the camera projection model. After the pattern of the barcode feature frame C has been located, the pixel coordinates of *P*′ and *Q*′ need to be determined to calculate the length of *P*′*Q*′ in the pixel coordinate system. *P*′ is determined to be the point that has the minimum sum of X and Y pixel coordinates out of all the points on the inner outline of the C pattern, while *Q*′ is the point with the largest ratio of the difference between its Y pixel coordinate to Y pixel coordinate of *P*′ and its X pixel coordinate. The pixel coordinates of *P*′ and *Q*′ can be calculated using the following formulas (3) and (4)

$$\left(x_{p},y_{p})=(x_{i},y_{i})|\text{min}(x_{i}+y_{i}\right)$$ (3)

$$\left(x_{q},y_{q})=(x_{i},y_{i})|\text{max}((y_{i}-y_{p})/x_{i}\right)$$ (4)

where, *x*_*i*_ and *y*_*i*_ are the X and Y pixel coordinates of a point on the inner outline of the C pattern, respectively. In Fig 8, a green circle and a green cross are used to indicate projection points *P*′ and *Q*′ in the CMOS imaging plane, respectively. Fig 9 presents the locating process of the barcode tag feature frame C pattern.

#### B. Recognition of barcode information

**Fig 10:**
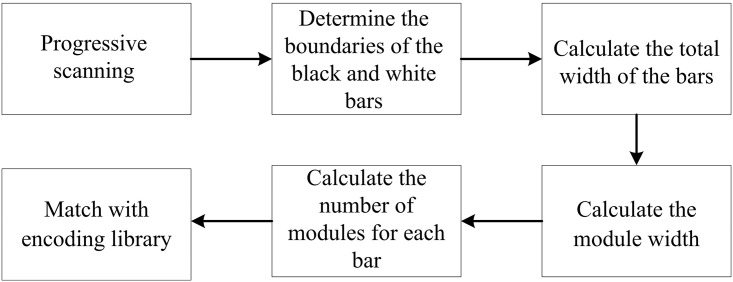
Recognition process of the encoded barcode information.

After the pattern of C is located in the images obtained by the cameras, progressive scanning is used to recognize the barcode within the pattern C in image *I*_*2*_. In the binary image *I*_*2*_, let the leftmost and the rightmost two pixels on the *i*^*th*^ row of the inner outline of the C pattern be *p*(*i*, *s*) and *p*(*i*, *r*). Then the coordinate values on the X axis of the pixels on the *i*^*th*^ row between *p*(*i*, *s*) and *p*(*i*, *r*) are within the range *s* < *j* < *r*. If any pixel *p*(*i*, *j*) and its neighboring pixel *p*(*i*, *j* + 1) have different values (0 or 1), these two pixels are the boundary points of the black and white bars. All boundary points on the *i*^*th*^ row can be determined in the same way. With these boundaries, the width *B*_*u*_ of each of the 59 black or white bars can be calculated. The total width of the 59 barcode bars is

$$S_{B}=\overset{59}{\underset{u=1}{\sum }}B_{u}$$ (5)

where, *u* is the number of barcode bars. The module width is *b* = *S*_*B*_ / 95. Each bar has a width of *c* = *B*_*u*_ / *b* module width. With the exception of the start symbol on the left, the end symbol on the right and the separation symbol in the middle, each set of 4 bars from the left to the right of the remaining bars are grouped together, and then match with predefined encoding rules to obtain the corresponding digital information. Progressive scanning of the content surrounded by the C pattern gives the coordinates of *P*(0.3m, 3m, 0.6m). Fig 10 shows the recognition process of the encoded barcode information.

## Dual cameras localization model and localization algorithm

**Fig 11:**
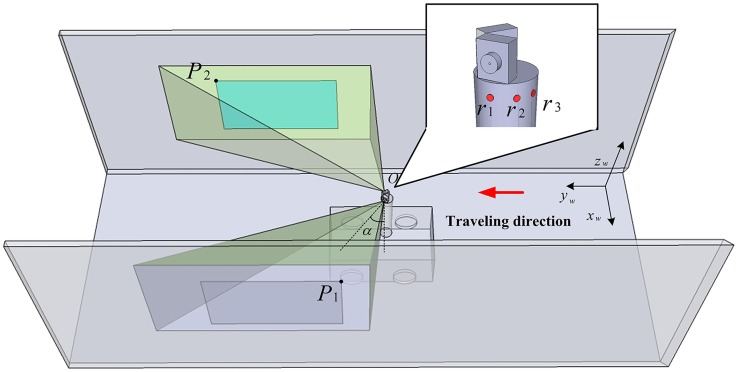
Vehicle dual camera autonomous localization illustration.

Fig 11 illustrates the vehicle autonomous localization in local area ofa coal mine tunnel using dual cameras. *P*_1_ and *P*_2_ are the upper left inner corner points of the feature frame C of the barcode tags affixed to both side walls respectively. In the same tunnel, the upper left inner corner points *P* of all barcode tag feature frames share the same value *H*_*p*_ on the *z*_*w*_ axis in the global coordinate system *o*_*w*_*x*_*w*_*y*_*w*_*z*_*w*_. As shown in Fig 11, two cameras are mounted side by side on the vehicle, and the angle between the optical axis and the cross section of the tunnel is *α*. Since the camera is quite small compared to the tunnel space, the two camera lenses can be considered as a single point *O*, which is also the center point of the vehicle. The coordinates of *O* can be denoted as (*x*_*wo*_, *y*_*wo*_, *z*_*wo*_) in the global coordinate system *o*_*w*_*x*_*w*_*y*_*w*_*z*_*w*_ and the height to the tunnel bottom can be denoted as *H*_*o*_. In the following experiments, three ultrasonic sensors *r*_1_, *r*_2_ and *r*_3_ are installed beneath the left camera to measure the distance from *O* to the tunnel wall on the left side. The effective measurement angle for each ultrasonic sensor is 40°. The measurement angle of the three ultrasonic sensors can reach almost 120°, which ensures accurate measurement of the distance even when vehicle is turning left or right. When the vehicle is traveling, the image capturing area of the camera is approximately a quadrangular pyramid. When the quadrangular pyramid completely covers a barcode tag on the wall, the camera can locate and recognize the barcode tag using the above locating and recognition algorithm. It can be assumed that when coal mine vehicleat a measurement point, the recognized upper left inner corner points *P*_1_ and *P*_2_ of feature frames of barcode tags affixed to the walls on both sides of the tunnel have global coordinates of *P*_1_ (*x*_*w*1_, *y*_*w*1_, *z*_*w*1_) and *P*_2_ (*x*_*w*2_, *y*_*w*2_, *z*_*w*2_), *z*_*w*1 =_ *z*_*w*2_ = *H*_*p*_, respectively.

### Distance measurement

**Fig 12:**
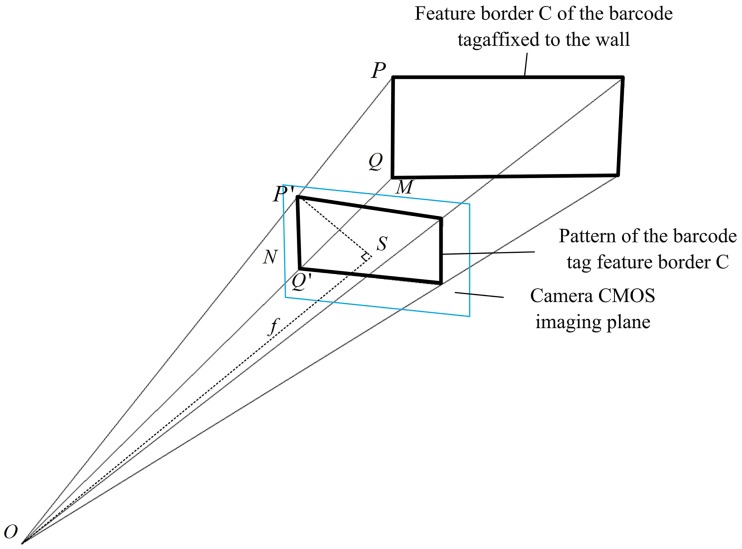
Projection model of the barcode tag feature frame C on the CMOS imaging plane.

For autonomous localization within the local area of tunnel, a traveling vehicle needs to measure the distances *D*_1_ and *D*_2_ from *O* to the upper left inner corner points *P* of C on the walls on both sides of the tunnel. Fig 12 shows the projection model of feature frame C of the barcode tag affixed to a tunnel wall in a camera CMOS imaging plane [36, 37]. In this figure, the blue rectangle is a CMOS imaging plane and *PQ* is the inner side of C with an actual length of *H*_*CI*_. The actual physical length of line segment from *P*′ (the projection point of *P*) to *Q*′ (the projection point of *Q*) on the CMOS imaging plane is

$$P\prime Q\prime =d_{pq}\times \frac{M}{m}$$ (6)

where, *d*_*pq*_ is the distance between *P*′ and *Q*′ in the CMOS imaging plane pixel coordinate system.

Due to the camera internal structure, the projection point *S* of the camera center *O* on the CMOS imaging plane is also the center point of the CMOS imaging plane. *OS* is the focal length of the camera with a length *f*. *SP*′ is the line segment between the projection point *P*′ and the center point *S*. Its actual physical length is

$$SP\prime =d_{sp}\times \frac{M}{m}$$ (7)

where, *d*_*sp*_ is the distance between *S* and *P*′ in the CMOS imaging plane pixel coordinate system.

Since *OS* ⊥ *SP*′, the length of *OP*′ can be calculated as shown below

$$OP\prime =\sqrt{SP\prime^{2}+OS^{2}}=\sqrt{{\left(d_{sp}\times \frac{M}{m}\right)}^{2}+f^{2}}$$ (8)

Since the triangle *OP*′*Q*′ and triangle *OPQ* are similar, we obtain

$$\frac{OP\prime }{OP}=\frac{P\prime Q\prime }{PQ}$$ (9)

Then the distance *D* between *P* and *O* can be calculated using formulas (6), (8) and (9)

$$D=\frac{PQ\times \sqrt{{\left(d_{sp}\times \frac{M}{m}\right)}^{2}+f^{2}}}{d_{pq}\times \frac{M}{m}}$$ (10)

### Localization

**Fig 13:**
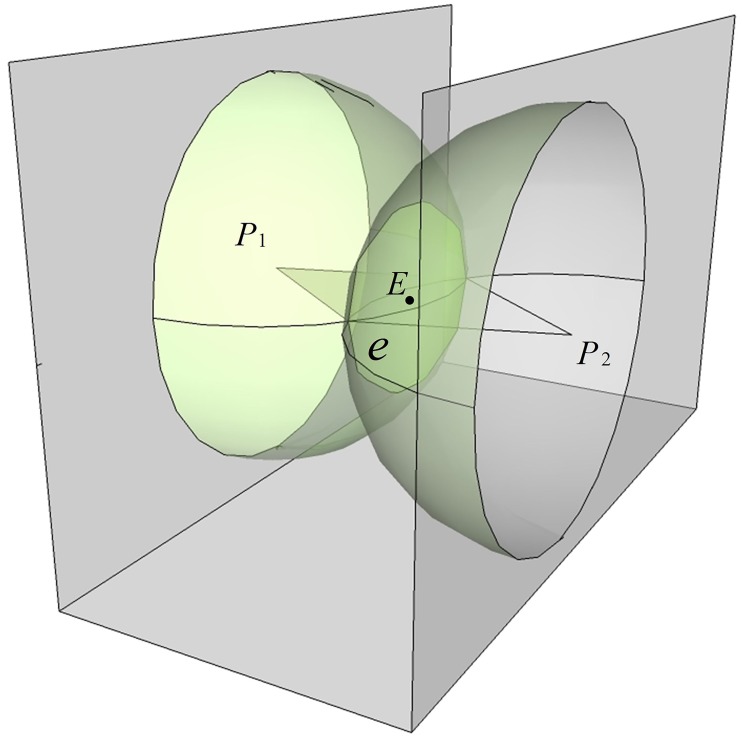
The circle *e* containing the vehicle center point *O*.

After obtaining the distances *D*_1_ and *D*_2_, the vehicle center point *O* is within the intersection circle *e* (with center point *E*) of the two spheres that have *P*_1_ and *P*_2_ as their centers and *D*_1_ and *D*_2_ as their radii, as shown in Fig 13.

**Fig 14:**
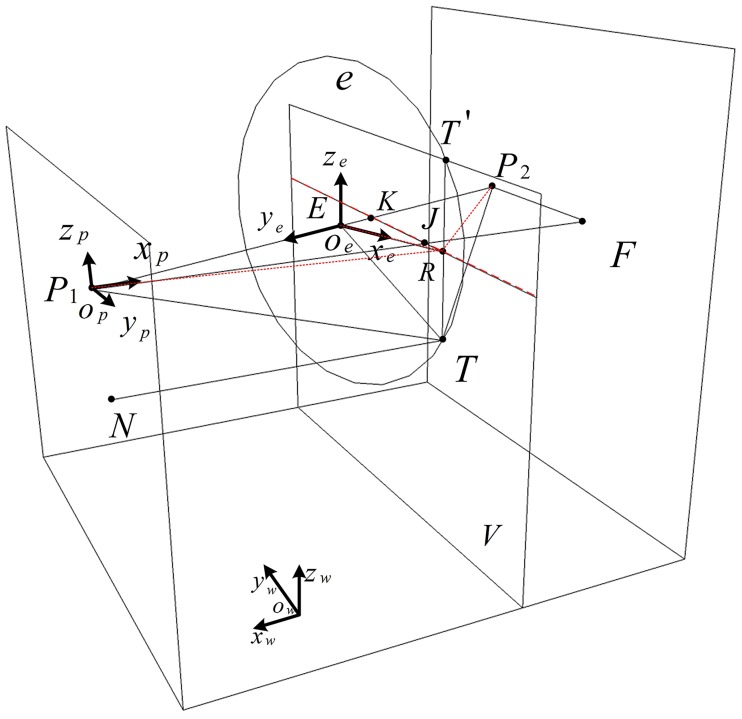
Spatial geometric diagram for the localization of the vehicle center point *O*.

To determine the position of the vehicle center point *O* within *e*, the relevant spatial geometric relationships within the tunnel are drawn as shown in Fig 14. In this figure, *o*_*w*_*x*_*w*_*y*_*w*_*z*_*w*_ is the global coordinate system of the tunnel. Since the barcode tags on the left and the right sides are symmetric, and *F* is the right upper inner corner point of C, *P*_1_*F* with length *W*_*T*_, is therefore perpendicular to the tunnel wall. Choosing the minimum distance of all distances between the vehicle center point *O* and left wall, which were measured respectively by the three ultrasonic sensors at the same time, as *d*. Do plane V at distance *d* from the wall on the left side. The plane V intersects with the circle *e* at points *T* and *T*′, which are the two candidates for the vehicle center point *O*. If *P*′ is above the CMOS imaging plane center *S*, i.e., the pixel coordinate *y*_*p*_>*n* / 2, then the vehicle center point *O* is *T*; otherwise, the center point is *T*′. *R* is the midpoint of *TT*′ in V.

In Fig 14, the coordinate system *o*_*p*1_*x*_*p*1_*y*_*p*1_*z*_*p*1_ is established with *P*_1_ as its origin, where the *x*_*p*1_ axis is perpendicular to the tunnel wall, the *y*_*p*1_ axis is along the longitudinal direction of the tunnel, and the *z*_*p*1_ axis is along the vertical direction of the tunnel. The coordinate system *o*_*e*_*x*_*e*_*y*_*e*_*z*_*e*_ is established with the center point *E* of the circle *e* as its origin, where the *x*_*e*_ axis is along the horizontal direction of the tunnel ground, the *y*_*e*_ axis is perpendicular to the plane of *e*, and the *z*_*e*_ axis is along the vertical direction of the tunnel.

The coordinates of *T* and *T*′ are firstly calculated on the *z*_*w*_ axis in the global coordinate system *o*_*w*_*x*_*w*_*y*_*w*_*z*_*w*_. In Fig 14, the equation for the plane V in the coordinate system *o*_*p*1_*x*_*p*1_*y*_*p*1_*z*_*p*1_ is *x*_*p*1_ = *d*. The two points *K*(*x*_*kp*_, *y*_*kp*_, *z*_*kp*_) and *J*(*x*_*jp*_, *y*_*jp*_, *z*_*jp*_) can be selected on V. They are the intersection points of *P*_1_*P*_2_ and *P*_1_*F* with V, respectively. Therefore, their coordinates in the coordinate system *o*_*p*1_*x*_*p*1_*y*_*p*1_*z*_*p*1_ are (*d*,−*d*tan*θ*,0) and (*d*,0,0), respectively. The transformation relationship from the coordinate system *o*_*p*1_*x*_*p*1_*y*_*p*1_*z*_*p*1_ to the coordinate system *o*_*e*_*x*_*e*_*y*_*e*_*z*_*e*_ is:

$$\left[\begin{array}{c} x_{e}\\ y_{e}\\ z_{e} \end{array}\right]=R_{p1e}\times \left[\begin{array}{c} x_{p1}\\ y_{p1}\\ z_{p1} \end{array}\right]+T_{p1e}$$ (11)

where, *R*_*p*1*e*_ is the rotation matrix from the coordinate system *o*_*p*1_*x*_*p*1_*y*_*p*1_*z*_*p*1_ to the coordinate system *o*_*e*_*x*_*e*_*y*_*e*_*z*_*e*_, and *T*_*p*1*e*_ is a translational matrix.

$$R_{p1e}=\left[\begin{array}{ccc} \text{cos}\left(\frac{\pi }{2}-\theta \right) & \text{sin}\left(\frac{\pi }{2}-\theta \right) & 0\\ -\text{sin}\left(\frac{\pi }{2}-\theta \right) & \text{cos}\left(\frac{\pi }{2}-\theta \right) & 0\\ 0 & 0 & 1 \end{array}\right],{\;T}_{p1e}=\left[\begin{array}{c} 0\\ P_{1}E\\ 0 \end{array}\right]$$

$$P_{1}E=\left(D_{1}{}^{2}-D_{2}{}^{2}+{\left(W_{T}/\text{cos}\theta \right)}^{2}\right)/2(W_{T}/\text{cos}\theta ).$$

Therefore, the coordinates of *K* and *J* in the coordinate system *o*_*e*_*x*_*e*_*y*_*e*_*z*_*e*_ can be calculated using formula (11)

$$\left[\begin{array}{c} x_{ke}\\ y_{ke}\\ z_{ke} \end{array}\right]==\left[\begin{array}{c} 0\\ P_{1}E-\left(d\text{cos}\theta +d\text{sin}\theta \text{tan}\theta \right)\\ 0 \end{array}\right]$$ (12)

$$\left[\begin{array}{c} x_{je}\\ y_{je}\\ z_{je} \end{array}\right]=\left[\begin{array}{c} d\text{sin}\theta \\ P_{1}E-d\text{cos}\theta \\ 0 \end{array}\right]$$ (13)

After obtaining the coordinates of *K* and *J* in the coordinate system *o*_*e*_*x*_*e*_*y*_*e*_*z*_*e*_, the equation of V in *o*_*e*_*x*_*e*_*y*_*e*_*z*_*e*_ can be calculated as follow

$$y_{e}=\text{tan}\theta x_{e}+a$$ (14)

where, *a* = *P*_1_*E* − (*d* cos *θ* + *d* sin *θ* tan *θ*).

In the coordinate system *o*_*e*_*x*_*e*_*y*_*e*_*z*_*e*_, the equation for circle *e* is

$$\{\begin{array}{l} r^{2}=x_{e}{}^{2}+z_{e}{}^{2}\\ y_{e}=0 \end{array}$$ (15)

where, *r* is the radius of *e*, and $r^{2}=D_{1}^{2}-{P_{1}E}^{2}$.

Therefore, the coordinates of *T* and *T*′ in the coordinate system *o*_*e*_*x*_*e*_*y*_*e*_*z*_*e*_ can be calculated using formulas (14) and (15) as shown below

$$\left[\begin{array}{c} x_{te}\\ y_{te}\\ z_{te} \end{array}\right]=\left[\begin{array}{c} -a/\text{tan}\theta \\ 0\\ -\sqrt{r^{2}-{\left(a/\text{tan}\theta \right)}^{2}} \end{array}\right]$$ (16)

$$\left[\begin{array}{c} x_{t\prime e}\\ y_{t\prime e}\\ z_{t\prime e} \end{array}\right]=\left[\begin{array}{c} -a/\text{tan}\theta \\ 0\\ \sqrt{r^{2}-{\left(a/\text{tan}\theta \right)}^{2}} \end{array}\right]$$ (17)

Since the *z*_*w*_ axis of the global coordinate system *o*_*w*_*x*_*w*_*y*_*w*_*z*_*w*_ is in the same direction as the *z*_*e*_ axis of the coordinate system *o*_*e*_*x*_*e*_*y*_*e*_*z*_*e*_, and the upper left inner corner point *P* of feature frame C in the global coordinate system *o*_*w*_*x*_*w*_*y*_*w*_*z*_*w*_ has a coordinate value *H*_*p*_ on the *z*_*w*_ axis, the coordinate values on the *z*_*w*_ axis of *T* and *T*′ in the global coordinate system are

$$z_{tw}=H_{p}-\sqrt{r^{2}-{\left(a/\text{tan}\theta \right)}^{2}}$$ (18)

$$z_{t\prime w}=H_{p}+\sqrt{r^{2}-{\left(a/\text{tan}\theta \right)}^{2}}$$ (19)

After obtaining these coordinate values, the coordinate value of *z*_*wo*_ on the *z*_*w*_ axis of the vehicle center point *O* in the global coordinate system *o*_*w*_*x*_*w*_*y*_*w*_*z*_*w*_ can be calculated based on the relationship between pixel coordinate *y*_*p*_ of *P*′ and *n* / 2.

In Fig 14, *R* is the midpoint of *TT*′. The coordinate values of *R* on the *x*_*w*_ and *y*_*w*_ axis in the global coordinate system share the same coordinate values as *T* or *T*′ on the *x*_*w*_ axis and *y*_*w*_ axis, respectively. The *x*_*e*_ of *R* in the coordinate system *o*_*e*_*x*_*e*_*y*_*e*_*z*_*e*_ is −*a* / tan *θ*, so the length of *ER* is −*a* / tan *θ*. Since *ER* ⊥ *P*_1_*P*_2_, the lengths of *P*_1_*R* and *P*_2_*R* respectively are

$$P_{1}R^{2}={\left(a/\text{tan}\theta \right)}^{2}+P_{1}E^{2}$$ (20)

$$P_{2}R^{2}={\left(a/\text{tan}\theta \right)}^{2}+P_{2}E^{2}$$ (21)

where, *P*_2_*E* = *W*_*T*_/cos *θ* − *P*_1_*E*. Since

$$P_{1}R^{2}={\left(x_{tw}-x_{p1}\right)}^{2}+{\left(y_{tw}-y_{p1}\right)}^{2}$$ (22)

$$P_{2}R^{2}={\left(x_{tw}-x_{p2}\right)}^{2}+{\left(y_{tw}-y_{p2}\right)}^{2}$$ (23)

the two plane coordinate values (*x*_*tw*_,*y*_*tw*_) and (*x*_*tw*_′,*y*_*tw*_′), where *y*_*tw*_ < *y*_*tw*_′, of *T* and *T*′ in the global coordinate system *o*_*w*_*x*_*w*_*y*_*w*_*z*_*w*_ can be calculated based on formulas (22) and (23). As the camera is placed obliquely forward on the center of the vehicle, the coordinates (*x*_*tw*_,*y*_*tw*_) that have a smaller value on the *y*_*w*_ axis are the coordinates of *T* or *T*′ in the global coordinate system *o*_*w*_*x*_*w*_*y*_*w*_*z*_*w*_, according to the geometric relationship shown in Fig 14, i.e., the plane coordinates of the vehicle center point *O* in the global coordinate system is (*x*_*wo*_,*y*_*wo*_).

## Tests and performance evaluation

### Tests in a straight corridor

**Fig 15:**
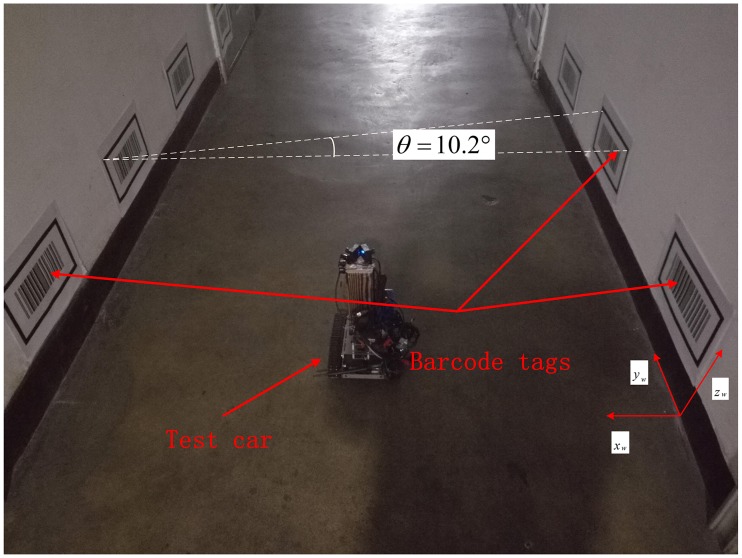
The physical corridor environment.

In order to evaluate the performance of the proposed autonomous localization method for coal mine vehiclein local area using double vision sensors to recognize barcode tags on both sides of tunnel walls with the help of ultrasonic sensors, this method was first tested in a straight corridor that simulates a straight tunnel. The corridor under test had a length of 10m, a width *W*_*T*_ of 1.9m, and a height of 3m, as shown in Fig 15. The global coordinate system *o*_*w*_*x*_*w*_*y*_*w*_*z*_*w*_ was established with the *x*_*w*_ axis, *y*_*w*_ axis and *z*_*w*_ axis along the longitudinal, horizontal and vertical directions of the corridor respectively. Eight pairs of barcode tags were affixed to the walls on both sides of the corridor. The interval distance *W*_*B*_ between any two neighboring barcode tags on the same side was 0.4m. The length *L* and width *H* of the tested barcode tags were 0.4m and 0.3m respectively. The actual physical lengths of the inner sides *L*_*CI*_ and *H*_*CI*_ of the feature frame C were 0.343m and 0.168m respectively. The upper left inner corner point *P* of the frame C was 0.4m high from the ground. The angle *θ* between the line from the upper left inner corner point *P* to the corner point *P* of the adjacent barcode on the opposite side of the wall and the line which is perpendicular to the corridor wall is 10.2°.

**Fig 16:**
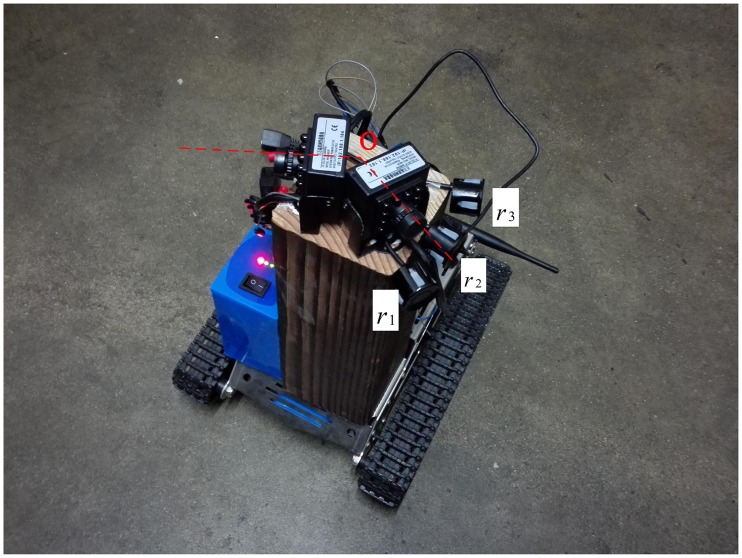
Test car.

The vehicle used in the experiments was a lithium-battery powered crawler car that could be manipulated by mobile phones through WIFI. The car was capable of spinning on the spot, as shown in Fig 16. Two TY803-130 infrared cameras were installed on the test car. The angles *α* between the optical axes of the two cameras and the perpendicular line to the corridor wall were both 35°. The relevant parameters of the infrared camera are shown in Table 2. The height of the vehicle center point *O* from the ground was 0.24m. Three JSN-SR04T ultrasonic sensors *r*_1_, *r*_2_ and *r*_3_ were mounted on the pillar beneath the left camera. Their data was read using an Arduino DUE development board. Ultrasonic sensors were used to transmit ultrasonic signals to measure the distance every 200ms. The relevant parameters are shown in Table 3.

**Table 2:** Camera parameters.

Parameters	Values
*f*/mm	6.0
*m*×*n*/pixel	1280×960
CMOS *M*×*N/mm*	4.8×3.6

**Table 3:** Ultrasonic sensor parameters.

Parameters	Values
Maximum range/cm	500
Minimum range/cm	25
Measuring angle/°	40

**Fig 17:**
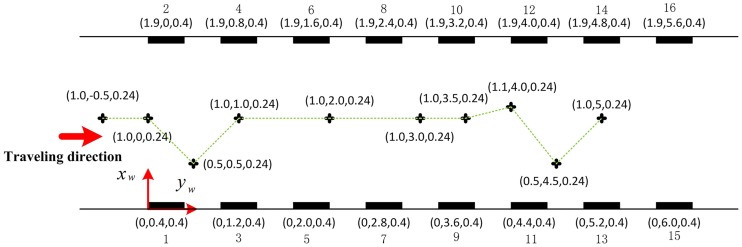
Topology diagram under test in the corridor.

**Fig 18:**
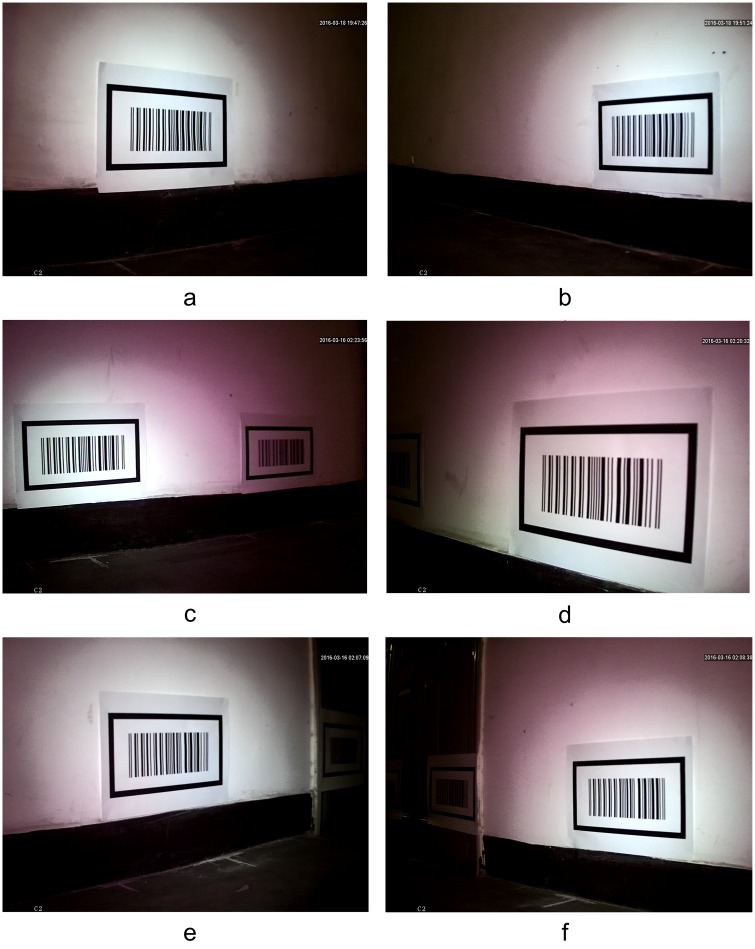
The original images captured by the on-board cameras at three of the measurement points. (a) The image captured at (1, -0.5) on the left side; (b) The image captured at (1, -0.5) on the right side; (c) The image captured at (0.5, 4.5) on the left side; (d) The image captured at (0.5, 4.5) on the right side; (e) The image captured at (1, 5) on the left side; (f) The image captured at (1, 5) on the right side;

Fig 17 shows a topology diagram of the test in the corridor, with the black rectangles on both sides showing the barcode tags. In the experiment, the car started from coordinates (1, -0.5) and traveled along the green track until it reached coordinates (1,5). The on-board cameras took 20 pictures in total when the car passed the 10 measurement points marked by crosses. Fig 18 shows the images captured by dual cameras at three of the measurement points at (1, -0.5), (0.5, 4.5) and (1, 5).

**Fig 19:**
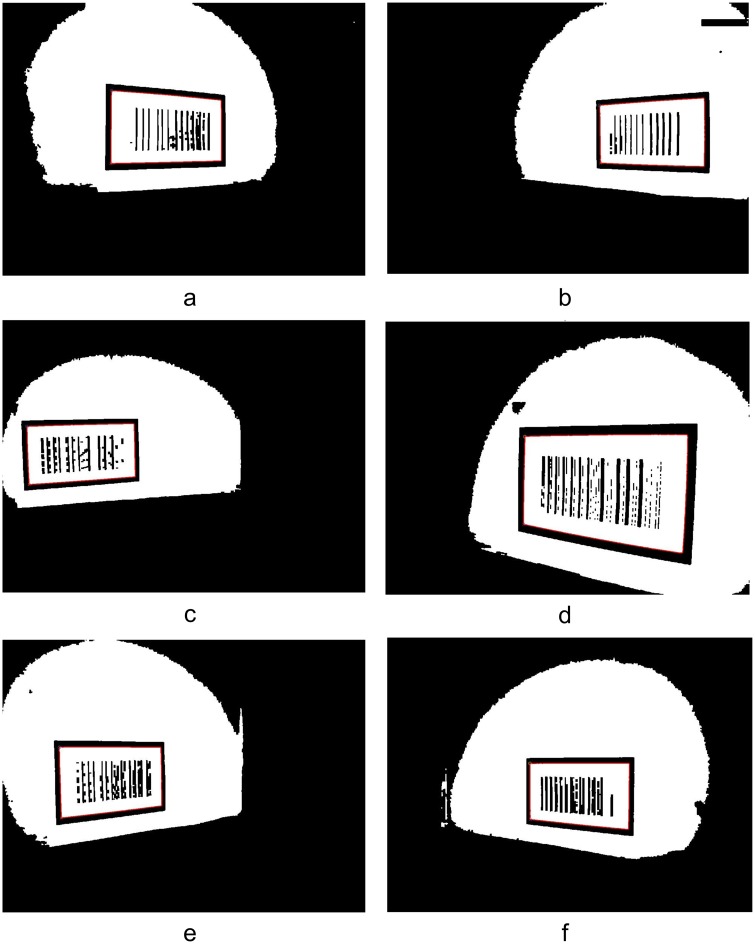
Locating results of the patterns of barcode tag feature frame C. (a) Locating of the feature frame C patterns on left side at (1,-0.5); (b) Locating of the feature frame C patterns on right side at (1,-0.5); (c) Locating of the feature frame C patterns on left side at (0.5,4.5); (d) Locating of the feature frame C patterns on right side at (0.5,4.5); (e) Locating of the feature frame C patterns on left side at (1,5); (f) Locating of the feature frame C patterns on right side at (1,5);

Fig 19 shows the locating results for the images in Fig 18 using the proposed barcode tag locating algorithm at the measurement points (1, -0.5), (0.5, 4.5) and (1, 5). From Fig 19, we can see that the proposed algorithm can effectively locate the pattern of the feature frame C of the barcode tags. A progressive scanning method was then used to recognize the barcode and obtain the coordinates of the upper left inner corner point *P* in the global coordinate system.

After the feature frame pattern was located and the barcode was recognized, the pixel coordinates of the projection points *P*′ and *Q*′ of the upper left inner corner point P and the lower left inner corner point Q on the CMOS imaging plane could be calculated for each pair of barcode tags. The distances *D*_1_ and *D*_2_ from the two upper left inner corner points *P* of each pair of barcode tags to the vehicle center point *O* was calculated using formulas (6), (7) and (10). The coordinates of *O*, (*x*_*wo*_, *y*_*wo*_, *z*_*wo*_), in the global coordinate system were computed based on formulas (18), (19), (22) and (23), with the distance *d*, from *O* to the left wall, measured by the ultrasonic sensors.

To evaluate the accuracy of the localization of *O* using the proposed localization algorithm, the error of *z*_*w*_ axis localization in the global coordinate system was defined as

$$E_{z}(i)=\left|z_{o}(i)-z_{r}(i)\right|$$ (24)

where, *z*_*o*_(*i*) is the calculated coordinate of *O* on the *z*_*w*_ axis at the *i*^*th*^ measurement point and *z*_*r*_(*i*) is the actual coordinate of *O* on the *z*_*w*_ axis. Therefore, the average error of *z*_*w*_ axis localization is

$$\bar{E_{z}}=\frac{1}{U}\overset{U}{\underset{i=1}{\sum }}E_{z}(i)$$ (25)

Similarly, the error of plane coordinate localizationfor *O* in the global coordinate system can be defined as

$$E_{p}(i)=\sqrt{{\left(x_{o}(i)-x_{r}(i)\right)}^{2}+{\left(y_{o}(i)-y_{r}(i)\right)}^{2}}$$ (26)

where, *x*_*o*_(*i*) and *y*_*o*_(*i*) are the calculated coordinates of *O* on the *x*_*w*_ and *y*_*w*_ axis respectively at the *i*^*th*^ measurement point, while *x*_*r*_(*i*) and *y*_*r*_(*i*) are the actual coordinates respectively. For *U* measurement points, the average error of plane coordinate localization for *O* is

$$\bar{E_{p}}=\frac{1}{U}\overset{U}{\underset{i=1}{\sum }}E_{p}(i)$$ (27)

Table 4 shows the coordinates of the upper left inner corner points *P*_1_ and *P*_2_ of C in pairsrecognized at each of the ten measurement points in the global coordinate system *o*_*w*_*x*_*w*_*y*_*w*_*z*_*w*_, and their localization results and errors using the proposed algorithm.

**Table 4:** Localization results and errors.

Number	*P*_1_ (*x*_*w*1_, *y*_*w*1_, *z*_*w*1_)/m	*P*_2_ (*x*_*w*2_, *y*_*w*2_, *z*_*w*2_)/m	*d*/m	Localization results (*x*_*wo*_, *y*_*wo*_, *z*_*wo*_)/m	Errors *E*_p_, *E*_z_/m
1	(1.9,0,0.4)	(0,0.4,0.4)	0.902	(0.980,-0.419,0.151)	0.083,0.089
2	(1.9,0.8,0.4)	(0,1.2,0.4)	0.901	(0.964,0.175,0.201)	0.179,0.039
3	(1.9,1.6,0.4)	(0,1.2,0.4)	1.403	(0.517,0.769,0.183)	0.269,0.057
4	(1.9,1.6,0.4)	(0,2.0,0.4)	0.901	(0.983,1.230,0.112)	0.231,0.128
5	(1.9,2.4,0.4)	(0,2.8,0.4)	0.906	(0.984,2.208,0.053)	0.209,0.187
6	(1.9,3.2,0.4)	(0,3.6,0.4)	0.899	(0.988,2.906,0.198)	0.095,0.042
7	(1.9,4.0,0.4)	(0,4.4,0.4)	0.902	(0.979,3.500,0.233)	0.021,0.007
8	(1.9,4.8,0.4)	(0,5.2,0.4)	0.803	(1.065,3.951,0.132)	0.060,0.108
9	(1.9,4.8,0.4)	(0,5.2,0.4)	1.410	(0.467,4.358,0.210)	0.146,0.030
10	(1.9,5.6,0.4)	(0,6.0,0.4)	0.903	(0.975,5.121,0.096)	0.124,0.144

**Fig 20:**
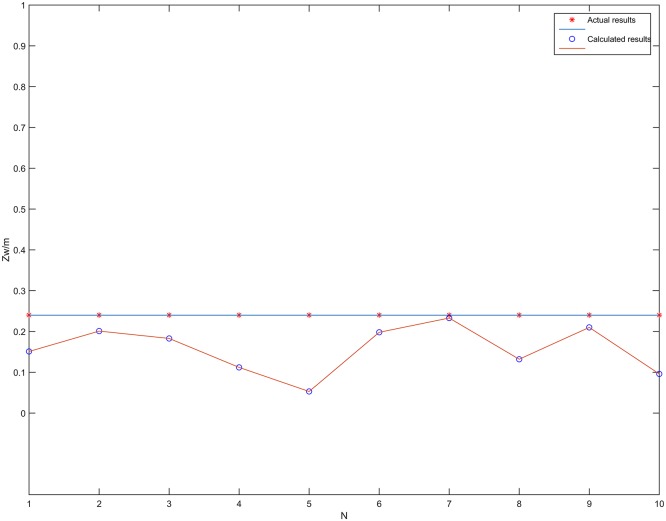
Localization results of the *z*_*w*_ axis coordinate of the vehicle center *O* in a straight corridor.

The localization results of the vehicle center point *O* on the *z*_*w*_ axis in Table 4 are plotted in Fig 20. From Fig 20, it can be seen that the proposed localization algorithm can effectively compute the coordinates of *O* on the *z*_*w*_ axis. The average error $\bar{E_{z}}$ on the *z*_*w*_ axis for the ten measurement points is 0.083m, calculated using formula (25). This shows that the proposed algorithm can quite accurately locate the *z*_*w*_ coordinate of the vehicle center *O* in a straight corridor.

**Fig 21:**
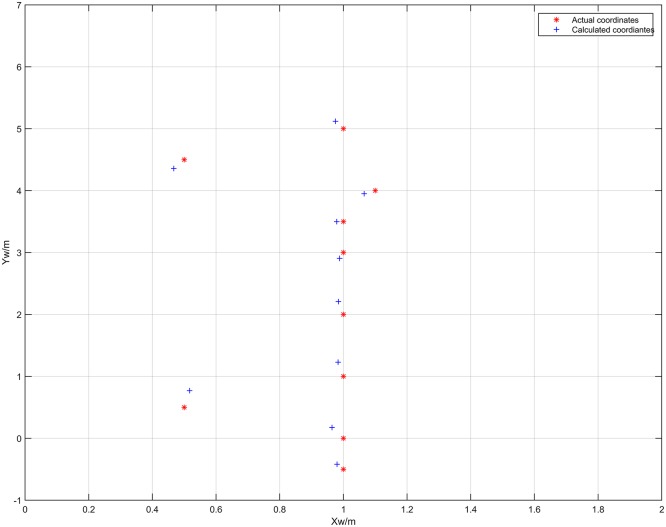
Results of the plane coordinate localization of the vehicle center point *O* in a straight corridor.

**Fig 22:**
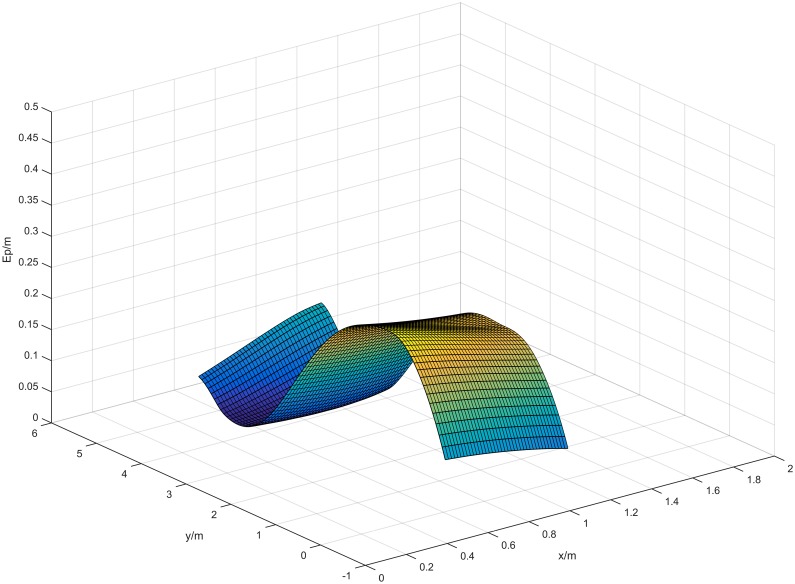
Error surface of plane coordinate localization of the vehicle center point *O* in a straight corridor.

Fig 21 shows the localization results of the plane coordinates of the vehicle center point, based on the results in Table 4. As can be seen from Fig 21, the proposed algorithm can also effectively compute the plane coordinates at each measurement point. Fig 22 shows the error surface of the plane coordinate localization, where *E*_*p*_ is the plane coordinate localization error of *O* at each measurement point. As shown in Fig 22, the localization error varies from 0.021m to 0.270m. When the values on the axes *x*_*w*_ and *y*_*w*_ reach 0.5m and 1m respectively, the error *E*_*p*_ reaches the maximum value which is 0.2695m. When the values on the axes *x*_*w*_ and *y*_*w*_ reach 1m and 4m respectively, the error *E*_*p*_ reaches the minimum value which is 0.021m. This is because when the experimental vehicle is at the coordinates (0.5, 0.5), the distance between the camera and the barcode tag on the right wall is minimum. Consequently the length of *P*′*Q*′ gets its maximum value, leading to a maximum error of distance *D*. In a similar way, when the experimental vehicle is at the coordinates (1.1, 4.0), the distances between the cameras and the barcode tag on the both sides of walls are relatively long. Consequently, the length of *P*′*Q*′ gets its minimum value, leading to a minimum error of distance *D*.

The average error $\bar{E_{p}}$ is calculated as 0.142m using formula (27). This result shows the accuracy of the proposed algorithm for locating the plane coordinates of the vehicle center point *O*.

### Tests in a curved tunnel

**Fig 23:**
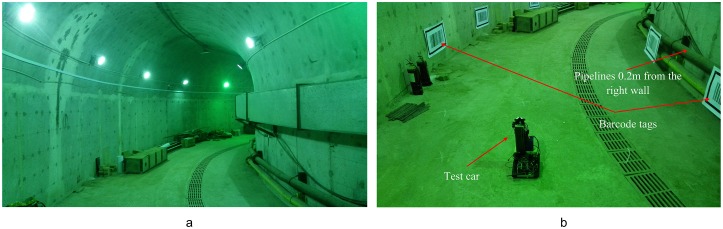
Test environment of the underground tunnel. (a) Real figure of the curved tunnel; (b) Deployment graph of the barcode tags;

The curved tunnel test site was the underground tunnel at the Research Center of Tunnel and Underground Engineering of Beijing Jiaotong University. The actual environment of tunnel was as shown in Fig 23(a) and 23(b). The cross-sectional shape of the tunnel was horseshoe-shaped with a width of 4m. Fig 23(b) shows six pairs of barcode tags symmetrically deployed on both sides of the tunnel. Since there were pipelines on the right tunnel wall, the barcode tags were affixed to the pipelines 0.2m from the right wall, so the actual tunnel width *W*_*T*_ was 3.8m. The length *L* and width *H* of the barcode tags in this experiment were 0.8m and 0.43m respectively. The lengths of the inner sides *L*_*CI*_ and *H*_*CI*_ of the feature frame C were 0.665m and 0.325m respectively. The upper left inner corner point *P* of C was at a height of 0.6m from the ground, and the angle *θ* between the line between the two upper left inner corner points and the line perpendicular to the tunnel wall was 9.93°.

In the curved tunnel test, the height *H*_*o*_ of the vehicle center point *O* from the ground was 0.35m; and the angle between the optical axis of the two cameras and the line perpendicular to the wall was 30°. The parameters of the cameras and the ultrasonic sensors are the same as the previous straight corridor experiment.

**Fig 24:**
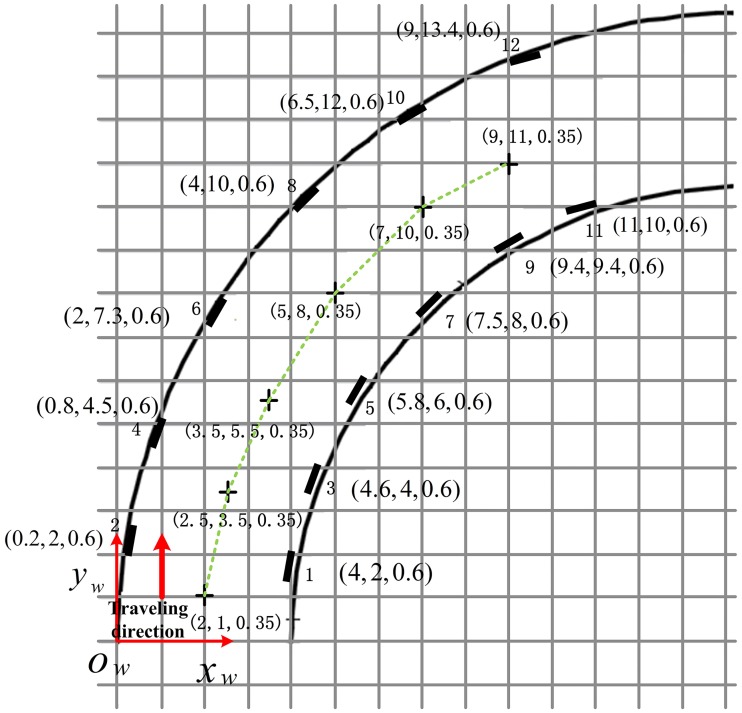
Experiment topology in the curved tunnel.

Fig 24 shows the experiment topology in the curved tunnel, with the black rectangles on both sides representing the barcode tags. A global coordinate system *o*_*w*_*x*_*w*_*y*_*w*_*z*_*w*_ was established at the entrance of the tunnel with the *x*_*w*_, *y*_*w*_ and *z*_*w*_ axis representing the longitudinal, horizontal and vertical directions of the tunnel entrance respectively.

**Fig 25:**
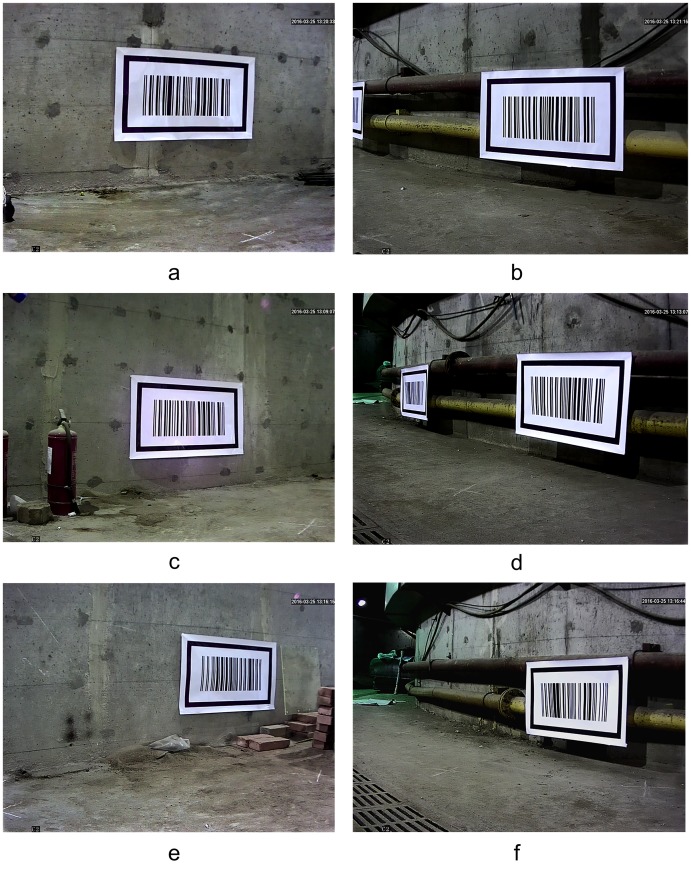
The original images collected at the three measurement points by the on-board cameras. (a) The images collected on left side at the measurement point (2,1); (b) The images collected on right side at the measurement point (2,1); (c) The images collected on left side at the measurement point (2.5,3.5); (d) The images collected on right side at the measurement point (2.5,3.5); (e) The images collected on left side at the measurement point (3.5,5.5); (f) The images collected on right side at the measurement point (3.5,5.5);

In this test, the vehicle started from the entrance coordinates (2,1), and traveled along the green track until coordinates (9,11). Twelve pictures were captured when passing the six measurement points, which were marked as crosses in Fig 24. Fig 25 shows the images that were collected by the cameras on both sides at three measurement points with coordinates (2, 1), (2.5, 3.5) and (3.5, 5.5).

**Fig 26:**
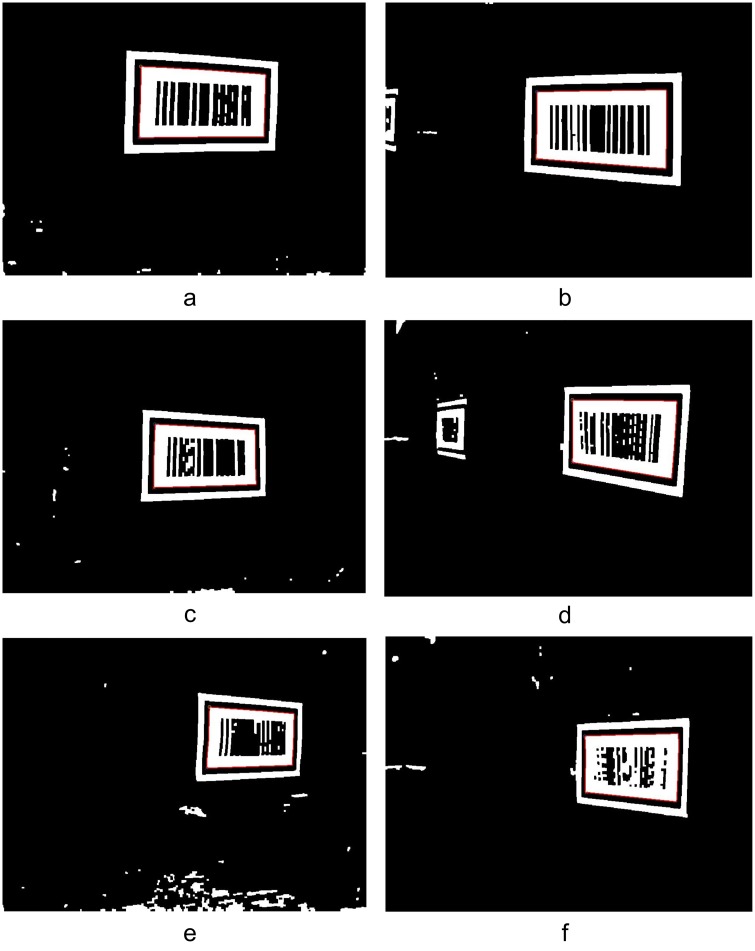
Locating results of the patterns of barcode tag feature frame C. (a) Locating of the feature frame C patterns on left side at (2,1); (b) Locating of the feature frame C patterns on right side at (2,1); (c) Locating of the feature frame C patterns on left side at (2.5,3.5); (d) Locating of the feature frame C patterns on right side at (2.5,3.5); (e) Locating of the feature frame C patterns on left side at (3.5,5.5); (f) Locating of the feature frame C patterns on right side at (3.5,5.5);

Fig 26 shows the locating results using the proposed algorithm for the images collected in Fig 25 at the measurement points (2,1), (2.5,3.5) and (3.5,5.5). Similarly, from Fig 26, it can be seen that the barcode locating and recognition algorithm can effectively locate the patterns of C. Progressive scanning was then also used to obtain the global coordinates of the upper left inner corner point *P* of C.

Similar to the straight corridor test, the cameras calculated the distances from the vehicle center point *O* to the upper left inner corner points of feature frame C on both sides of tunnel walls for each measurement point. The distance *d* from *O* to the left wall was then acquired from the ultrasound sensors can help to calculate the coordinates (*x*_*wo*_, *y*_*wo*_, *z*_*wo*_) of the vehicle center point *O* at each measurement point.

**Fig 27:**
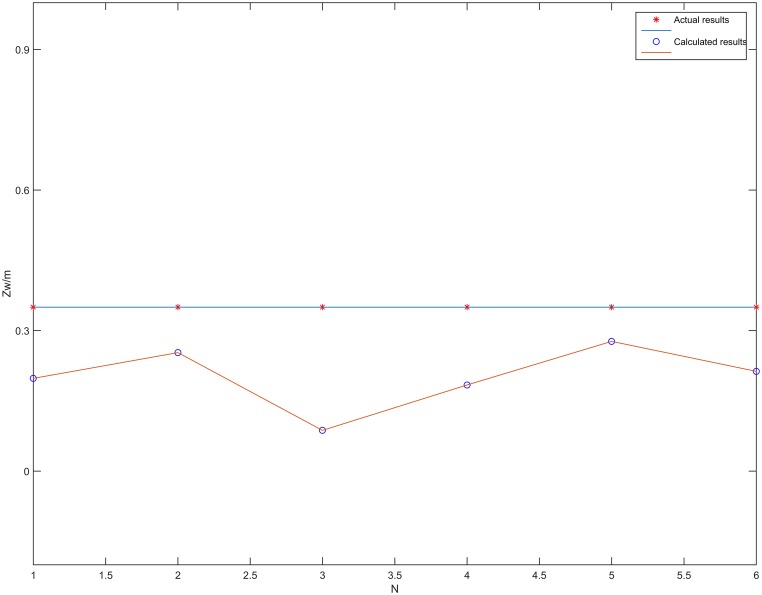
Localization results of the vehicle center point *O* on the *z*_*w*_ axis in the curved tunnel.

**Fig 28:**
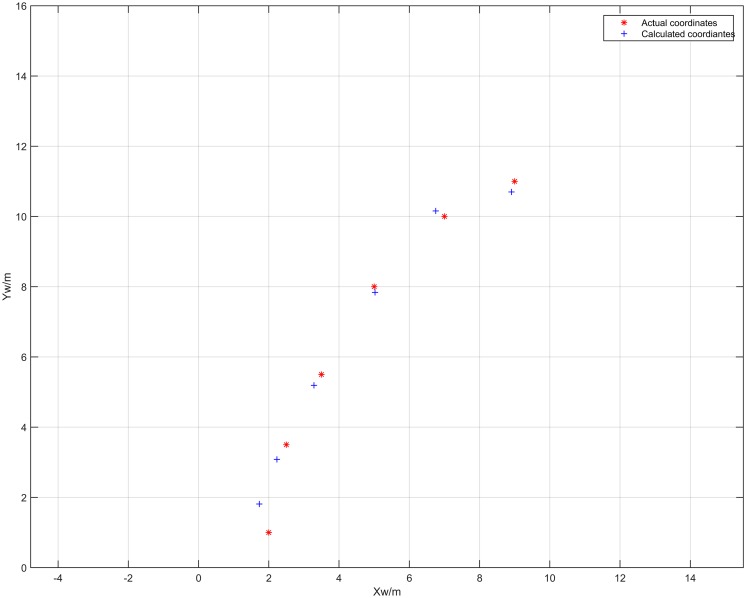
Results of the vehicle center point *O* plane coordinate localization in the curved tunnel.

Fig 27 shows the localization results of the coordinates in the *z*_*w*_ axis of *O* in the curved tunnel. Using formula (25), the average error of *z*_*w*_ axis localization of vehicle center point *O* for the six measurement points was 0.151m. Fig 28 shows the localization results of the plane coordinates for the six measurement points using the proposed algorithm. Using formula (27), the average error of plane coordinate localization was 0.381m. Therefore, the proposed localization algorithm is quite accurate and feasible in curved tunnels for both locating the vehicle center point on the *z*_*w*_ axis and locating the plane coordinates.

## Conclusions

For autonomous vehicles to travel in local area of a coal mine tunnel, a self-localization capability is required. We propose an autonomous localization method in local area of coal mine tunnel that uses on-board vision sensors to recognize barcode tags affixed to the tunnel walls in pairs and ultrasonic sensors to measure the distance to the tunnel walls.

1. Using the proposed barcode tag locating and recognition algorithm, the vehicle vision sensors effectively located the feature frame from the collected images containing barcode tags affixed to the tunnel walls on both sides and were able to recognize the barcode tags.
2. The spatial geometric relationship between the vehicle center point and the barcode tags was established based on the distance between the vision sensors and the upper left inner corner points of the barcode tag feature frame in pairs and the distance from the vehicle center point to the left side tunnel wall. The 3D coordinates of the vehicle center point could then be calculated in the tunnel’s global coordinate system.
3. For evaluation of the proposed algorithm, experimental results in a straight corridor have shown that average error of *z*_*w*_ axis localization is 0.08m for locating the vehicle center point and average error of plane coordinate localization is 0.142m for locating the vehicle center point. Experimental results in the curved underground tunnel have shown that error of *z*_*w*_ axis localization is 0.151m for locating the vehicle center point and average error of plane coordinate localization is 0.381m for locating the vehicle center point.
